# Preliminary Exploration of Obesity-Related Eating Behaviour Patterns in a Sample of Saudi Preschoolers Aged 2–6 Years through the Children’s Eating Behaviour Questionnaire

**DOI:** 10.3390/nu13114156

**Published:** 2021-11-20

**Authors:** Ali H. Al-Hamad, Aljohara M. Al-Naseeb, Maha S. Al-Assaf, Suzan A. Al-Obaid, Bandar S. Al-Abdulkarim, Pawel K. Olszewski

**Affiliations:** 1 Department of Pathology & Laboratory Medicine, King Abdulaziz Medical City, Riyadh 22490, Saudi Arabia; 2 Clinical Nutrition Department, King Fahad Medical City, Riyadh 11525, Saudi Arabia; aalnaseeb@kfmc.med.sa (A.M.A.-N.); msalassaf@moh.go.sa (M.S.A.-A.); sualobaid@kfmc.med.sa (S.A.A.-O.); Saffary_211@yahoo.com (B.S.A.-A.); 3 Department of Biological Sciences, University of Waikato, Hamilton 3240, New Zealand; pawel@waikato.ac.nz

**Keywords:** children’s eating behaviour questionnaire, CEBQ, obesity, factorial validation, children

## Abstract

Background: The Children’s Eating Behaviour Questionnaire (CEBQ) is used with parents to determine the characteristics of eating behaviour of their children and, consequently, children’s propensity to become obese. It has been successfully used mainly in Western countries, but not in Saudi Arabia. In this pilot study, we explored the use of the Saudi version of the CEBQ for preschool children aged 2–6 years in Saudi Arabia, and assessed the associations between eating behaviours and children’s age, gender and relative weight and parental weight. Methods: Parents of 200 Saudi preschool children in Riyadh completed the Saudi version of the CEBQ. Factor analyses on all CEBQ items were performed and differences between genders and age groups were examined. Correlations between children’s BMI z-scores and eating behaviours were analysed using linear regression. Results: The factor analysis revealed an eight-factor solution similar to the theoretical factor structure, with good internal reliability and acceptable correlations between subscales. Boys scored higher than girls on food responsiveness; no difference between age groups was found. Positive associations between BMI z-scores and ‘food approach’ subscales, food responsiveness, enjoyment of food and emotional overeating were found, while ‘food avoidant’ subscales, satiety responsiveness and slowness in eating had inverse relationships with BMI z-scores. Maternal BMI had a positive association with BMI z-scores and food responsiveness. Conclusion: The CEBQ is a valid psychometric tool that can be reliably used to assess eating behaviour characteristics in Saudi preschool children.

## 1. Background

According to the World Health Organisation [1], obesity among children is one of the most critical public health issues. It is reported that over 40 million children under the age of five years are overweight; the majority of these live in low- and middle-income countries. Obese children are at risk of developing high blood pressure, heart disease and diabetes [2], and they are more likely to be obese in adulthood [3,4].

As in the majority of developed countries, Saudi Arabia—which has witnessed significant changes in lifestyle as a consequence modernisation over the past three decades [5]—has a very high prevalence of obesity among children and adults. Al-Dossary et al. [6] reported that the prevalence of overweight was 19.0% and of obesity 23.3% in Saudi children. Despite this, there is a lack of a sufficient data on obesogenic behavioural traits in Saudi children [7], which points to an urgent need for studies analysing eating behavioural patterns in order to define the characteristics of consummatory behaviour in this regional population and to conceptualise effective obesity prevention strategies [8].

Importantly, children’s eating behaviour patterns develop as early as during infancy and at the preschool age, and they serve as the basis for consumption characteristics later in life [9]. Specific behavioural traits associated with obesity in children include low satiety responsiveness, emotional overeating, and eating too fast [10,11]. Already in the preschool period, individual differences in eating behaviours are prominent and they stem from heritability, parental food preferences, taste, smell, food availability/quality practices and a child’s adiposity [12]. Furthermore, one of the strongest risk factors for developing obesity among children is elevated body weight of parents [13].

Early detection of eating behaviour traits that promote obesity help identify at-risk families and introduce interventions. This detection is usually done using questionnaires completed by parents. These forms describe eating behaviour of children, and relate it to the probability of obesity. The questionnaires need to be standardised for the population of interest, and their validity and usability have to be assessed for each country they are used in. One of the most comprehensive instruments applicable to preschool children is termed the Children’s Eating Behaviour Questionnaire (CEBQ), developed and verified in the UK [14,15].

The CEBQ is a comprehensive assessment tool which relies on 35 questions which were formulated based on interviews with parents about the way their children eat. The items cover four dimensions indicating ‘food approach’ (enjoyment of food, food responsiveness, emotional overeating, and desire to drink) and four dimensions measuring ‘food avoidant’ behaviour (satiety responsiveness, slowness in eating, emotional undereating, and food fussiness) [14].

The two subscales, food responsiveness (FR) and enjoyment of food (EF), assess eating in response to environmental food cues. These behaviours have been found to become clearer as children get older [14,15]. The desire to drink (DD) scale detects increased desire for children to drink frequently, especially sugar-sweetened drinks [16]. Satiety responsiveness (SR) means how able a child is to control the amount of food eaten to fulfil energy needs [14,15]. High scores on the slowness in eating (SE) reflect a reduction in eating rate as a result of low interest and enjoyment of food [11,17]. Food fussiness (FF) is related to a rejection of a substantial amount of new and familiar foods, leading to an inadequate variety in a diet [18]. Finally, the emotional overeating (EOE) and emotional undereating (EUE) scales refer to an increase or a decrease in eating under negative emotions, such as anger or anxiety [19].

The CEBQ has been successfully used in many populations across the world, mainly in countries of Western-type eating habits, including the United Kingdom [14,15,20,21], the Netherlands [22,23], Portugal [24], Canada [25], and Sweden [26]. China [27] and Chile [28] are the few non-Western countries in which the CEBQ data have not been collected or analysed. Considering the growing obesity rates in Saudi Arabia and the lack of previous use of the CEBQ there, we found it pertinent to examine the factor structure and the reliability of the Saudi CEBQ in preschool children between 2 and 6 years of age. We also assessed that eating behaviours are associated with children’s age, gender and relative weight and with parental weight. The overarching goal of this study was to preliminarily explore the use of the CEBQ in a convenience sample of Saudi preschoolers attending one of the country’s largest medical centres in the capital, Riyad, and advise on measures that need to be undertaken in follow-up projects to validate CEBQ for use in the broad Saudi population.

## 2. Methods

### 2.1. Research Permits

This study was reviewed and approved by the Human Research Ethics Committee of The University of Waikato, New Zealand (12-224E). It was also accepted by the Institutional Review Board of King Fahad Medical City, Ministry of Health, Riyadh, Saudi Arabia. In addition, the approval of the Ministry of Higher Education, Saudi Arabia was granted before the beginning of the research.

### 2.2. Measures

The original English version of the CEBQ was translated into Arabic at the King Fahad Medical City centre in a standard procedure that follows the ISPOR recommendation and the final version of the document was approved by a government-authorised legal translator. As per the standardized translation process, which follows the protocol for cross-cultural adaptation [29], the questions were translated into Arabic by a translator and then translated back to English by another one. Any translation discrepancies were resolved by consensus in a group consisting of six professionals in biomedical sciences. The CEBQ consists of 35 items and covers eight factors of eating styles. Parents were asked to rate their child’s eating behaviour on a five-point Likert scale (never, rarely, sometimes, often, always; 1–5). The CEBQ was distributed together with a request letter. Additional questions about the child’s birth date, gender, chronic diseases, current weight and height, and about both parents’ weight, height, and educational level (primary school, high school, and college/university) were recorded on the case sheet. The survey contained a participant information sheet stating explicitly the participants’ right to withdraw from the study.

### 2.3. Body Mass Index

Body mass index (BMI) was calculated as BMI = Weight (in kg) / Height^2 (in metres) for parents and children. Each parent’s weight category was classified using the international cut-off points [30] as: ‘normal weight’ (BMI < 25), ‘overweight’ (BMI 25–29.9) and ‘obese’ (BMI ≥ 30). The U.S. Centres for Disease Control and Prevention (CDC) reference BMI data adjusted for age and sex [31] were used to classify weight category of children as ‘Underweight’ (less than the 5th percentile), ‘normal weight’ (5th percentile to less than the 85th percentile), ‘overweight’ (85th to less than the 95th percentile) and ‘obese’ (equal to or greater than the 95th percentile). For analysis purposes, the CDC reference data were also used to convert each child’s BMI to a standardised z-score. Epi Info 7 software was used to calculate the BMI z-score.

### 2.4. Overview of Procedures and Participants

The research took a place at King Fahad Medical City (KFMC) in Riyadh, the capital and the largest city (pop. > 5.5 million) of Saudi Arabia. KFMC is one of the largest health care facilities in the country and consists of a paediatric hospital, a general hospital, a maternity hospital and a rehabilitation hospital. It is funded by the government of Saudi Arabia to provide treatment for citizens from around the country. This study was set in a public medical centre in order to reach a large number of participants of diverse backgrounds. Only healthy children, or children under acute care, were allowed to participate. With the help of the Clinical Nutrition Department staff at KFMC in describing the study purpose to the participants, the questionnaire was distributed to KFMC visitors and staff with Saudi nationality. Participants were contacted in the waiting rooms, clinics, kindergartens and wards. A total of 400 questionnaires were handed out with envelopes. Parents of preschool children aged 2–6 years old were asked to complete them either home or at the hospital, put in the envelope, seal it and then return it to the department. Only the researcher had the right to open the envelope. The collection of questionnaires for this research, which was carried out from February 2013 to May 2013, produced a total of 238 participants (59.5% response rate). Children with asthma (N = 18) and diabetes (N = 4), as well as children with missing information or missing parental weight and height (N = 16), were excluded from the study.

This resulted in a sample of 200 children, 100 boys and 100 girls, aged 2–6 years old from 175 families. Each family had only one or two children within the specified age group. The mean age of the children in the study was 4.1 years. The children were subdivided into four groups: 2 years (N = 56), 3 years (N = 40), 4 years (N = 35) and 5–6 years (N = 69). The questionnaire was completed by the fathers of 10.9% of the children, by the mothers for 82.9% and in 6.2% of the cases, both parents filled it out.

Parental education levels were divided into three groups; only 4% of the fathers and 4.5% of the mothers had a primary school or no education. The majority of the whole sample had a college/university education: 65.1% of the fathers and 66.9% of the mothers. The remaining 30.9% of the fathers and 28.6% of the mothers had a high school education level.

This cross-sectional study confirms that there is a high proportion of obesity and overweight among adults in Saudi Arabia. Across 175 families, 34.3% of fathers were obese, 48% overweight and only 17.7% were of normal weight. The mean BMI for fathers was 29.0 (SD 4.9, range 16.9–49.6).

The prevalence of obesity among mothers was similar to that of fathers. A total of 34.3% of mothers were categorised as obese, 38.2% as overweight and 27.5% as of normal body weight. The mean BMI for mothers was 28.2 (SD 5.1, range 17.1–44.6). In the whole sample of 200 preschool children aged 2–6 years, 72% of children were of normal weight, 18.5% were overweight and 9.5% obese.

Similar to the Swedish study on the CEBQ [26], children were classified into two groups depending on various parental weight categories to assess the influence of the parents’ weight on their children’s weight and eating behaviours: the first category included those with a minimum of one obese parent or two overweight parents, whereas the second category consisted of children with two normal-weight parents or one normal-weight and one overweight parent. Table 1 presents the demographic and anthropometric characteristics of the whole sample.

### 2.5. Statistical Analysis

In this preliminary study, we followed the analyses done in similar, recently published reports exploring validity of the CEBQ in select geographic locations (e.g., in the Swedish and Vietnamese cohorts [26,32]). In order to verify the underlying structure of the Arabic version of the questionnaire and determine whether it was similar to the original CEBQ [14], a Principal Components Analysis (PCA) with Direct oblimin rotation was performed on all thirty-five CEBQ items. In accordance with Svensson et. al. [26], a factor loading of 0.4 was set as the threshold loading for factor analysis. Internal reliability coefficients (Cronbach’s alpha) and (average) inter-item correlations were estimated for each factor identified from the evaluation of the CEBQ. Cronbach’s alpha is a good measure of internal consistency, and acceptable values are normally above 0.70 [33]. However, Cronbach’s alpha values near 0.60 can still be acceptable [34]. In terms of inter-item correlations, the recommended average inter-item correlation should fall in the range of 0.15–0.50 [35]. Mean scores were computed for each factor and the correlations between the eight subscales of the CEBQ were determined by Pearson’s correlations. Cohen’s guidelines [36] were used to interpret strengths of correlations between factors. (i.e., correlation between 0.5 and 1.0 is described as a “strong” effect size, between 0.3 and 0.5 as “moderate” and between 0.1 and 0.3 as “weak”). Eight subscales of the CEBQ were compared based on gender and parental combined weight groups using an independent *t*-test, and—based on the age group—using a one-way analysis of variance (ANOVA). A hierarchical multiple linear regression analysis was carried out to examine the association between children’s BMI z-scores as a continuous dependent variable and each eating behaviour subscale, controlling for age, gender, parental educational level and parental combined weight groups. Finally, Pearson’s correlation was tested between the CEBQ scales and maternal BMI, paternal BMI and children’s BMI z-scores. All statistical analyses were carried out using SPSS Statistics 18.0.

## 3. Results

### 3.1. Factor Analysis

The factor analysis revealed an eight-factor solution and confirmed the original eight-factor structure. Each of the eight scales had one factor with an eigenvalue of more than one. The eight factors accounted for 60.17% of the total variance. Items loaded onto each factor ranged from 2–6 items (Table 2).

Most of the scale items loaded as expected but a few items warrant attention. Four items did not load as expected compared to the original study. Firstly, the item ‘My child eats more when anxious’ did not load onto the expected factor Emotional over-eating (EOE), but on Emotional under-eating (EUE). Secondly, the item ‘My child decides that s/he doesn’t like a food, even without tasting it’, which loaded unto Food fussiness (FF) in the original study, now loaded onto the Satiety responsiveness (SR). Another item that did not load as expected was ‘My child eats more when s/he has nothing else to do’. This item loaded onto the Food responsiveness (FR) not onto the EOE. The item ‘My child is always asking for food’ loaded onto the Enjoyment of food (EF) factor, but not onto its original FR factor.

Only three items loaded onto two factors. First of all, the item ‘My child enjoys a wide variety of foods’ loaded most highly onto the FF factor (0.53), to which it originally belonged, but was also loaded onto the EF factor (0.43). The item ‘My child is difficult to please with meals’ originally belonging to the FF scale loaded onto FF and also FR, with a similar loading of 0.49 and 0.48 respectively. The item ‘My child eats less when s/he is tired’ loaded on the EUE scale on theoretical grounds (0.53) but also loaded onto the FR scale (0.42).

The three items ‘My child has a big appetite’ (SR), ‘My child looks forward to mealtimes’ (EF) and ‘Even if my child is full, s/he finds room to eat his/her favourite food’ (FR) loaded less than 0.4 and, thus, they were omitted to optimise the analysis [26].

### 3.2. Internal Reliability

The CEBQ subscales showed good internal reliability with Cronbach’s alpha ranging between 0.62 and 0.80. In addition, all the average inter-item correlations of the scales are considered ‘good’, ranging from 0.25 to 0.57 [33]. Table 3 presents the results of Internal reliability coefficients (Cronbach’s alpha) and (average) inter-item correlations for the eight scales of the instrument.

A few items were omitted in order to improve reliability of the scales. For instance, the omission of item ‘My child finishes his/her meal quickly’ from factor Slowness in eating (SE) increased the Cronbach’s alpha from 0.17 to 0.71. Cronbach’s alpha of factor Food Fussiness (FF) increased from 0.01 to 0.68 after ‘My child refuses new foods at first’ and ‘My child is difficult to please with meals’ were excluded. The items ‘My child eats less when s/he is tired’ and ‘My child is difficult to please with meals’ when excluded from factor Food responsiveness (FR) improved the Cronbach’s alpha from 0.52 to 0.76. Whenever omission of an item leads to an improvement in the internal consistency of a scale, it means that the omitted items do not measure the same construct as the rest of other items in the scale, and thus, it had to be excluded to improve the internal consistency of the scale [37].

### 3.3. Correlations between Scales

The correlations between scales (Table 4) suggest that the ‘food approach’ subscales (EOE, FR, EF and DD) and the two ‘food avoidant’ subscales (SR and SE) tend to be negatively inter-correlated, which is similar to previous reports [14,22,26]. In addition, moderate and positive correlations were found between the ‘food approach’ subscales (EOE, FR, EF and DD), while ‘food avoidant’ subscale EUE had weak positive correlations with all ‘food avoidant’ and ‘food approach’ subscales. The ‘food avoidant’ subscale FF tend to have positive inter-correlations with ‘food approach’ subscales and negative correlations with ‘food avoidant’ subscales. The highest correlation between any subscale was between FF and EF (r = 0.55).

### 3.4. Age, Gender and Combined Parental Weight Differences

The result showed that effect of age on eating behaviours is not significant. Likewise, gender has no significant effect on eating behaviours except on Food responsiveness (FR): boys scored higher than girls on FR (mean 2.2 (SD 1.1) versus 1.9 (SD 0.9), *p* = 0.04). Table 5 presents the gender and age differences in eating behaviour. The differences in children’s eating behaviour by parental combined weight groups are illustrated in Table 6. FR was also the only factor that significantly differed between the parental combined weight groups. The group of at least one obese parent or two overweight parents scored higher than the group of two normal-weight parents or one normal-weight and one overweight parent on FR (mean 2.1 (SD 1.0) versus 1.8 (SD 0.8), *p* = 0.02).

### 3.5. Correlations between BMI Z-Scores and Eating Behaviours

Regression analyses revealed no significant associations between children’s BMI z-scores and eating behaviour subscales, when controlled for gender, age, parental combined weight groups and parental educational level. Control variables, such as child gender, age, combined parental weight, maternal and paternal education level, and paternal and maternal BMI constrained into the models prior to adding each of the CEBQ scales revealed standardised β coefficients (*p*-values) of 0.052 (*p* = 0.446), 0.023 (*p* = 0.729), 0.187 (*p* = 0.054), −0.011 (*p* = 0.887), −0.087 (*p* = 0.230), −0.088 (*p* = 0.247) and 0.220 (*p* = 0.009), respectively. Only maternal BMI significantly contributed to the model. The result of the regression analysis to model the subscales of the CEBQ is presented in Table 7.

On the other hand, we identified significant positive correlations between child BMI z-scores and the ‘food approach’ factors EOE (r = 0.16, *p* = 0.02), FR (r = 0.23, *p* = 0.001) and EF (r = 0.24, *p* = 0.001). BMI z-scores had a linear decrease with ‘food avoidant’ subscales SR (r = −0.17, *p* = 0.016) and SE (r = −0.16, *p* = 0.019).

The correlation between child BMI z-scores and maternal BMI was positive and significant (r = 0.31, *p* = 0.009) and FR (r = 0.15, *p* = 0.03). Paternal BMI did not show any correlation with child BMI z-scores or any CEBQ subscales. Table 8 presents the correlations between CEBQ subscales and children’s BMI z-scores, maternal BMI and paternal BMI.

## 4. Discussion

This study is the first attempt to date to apply the Children’s Eating Behaviour Questionnaire (CEBQ) instrument in a sample of Saudi preschool children aged 2–6 years. It shows that the Saudi version of the CEBQ has good psychometric attributes with regard to factor structure, internal reliability and correlation between subscales, which is similar to the outcomes of validation studies in other geographic locations [14,22,24,26].

This study reveals an eight-factor structure which conforms to the original UK structure [14] and explains 60.17% of the variance. Although the original CEBQ had 35 items, only 31 items are presented here (Table 2). The four missing items—‘My child enjoys eating’, ‘My child looks forward to mealtimes’, ‘My child has a big appetite’ and ‘Even if my child is full up s/he finds room to eat his/her favourite food’—had a loading below 0.4 and were, therefore, excluded. Furthermore, the few items that did not load onto the expected original factors were removed from the analyses to improve the internal reliability of the scales. The final eight-factor structure of the Saudi version of the CEBQ was comparable with the original UK study and with other recent validation studies [14,22,24,26].

No age effects were observed in our cohort. This result is similar to the finding of the Dutch version of the CEBQ [22], but in contrast to the original and other validation studies [14,20,26]. The disparity between the result of this study and that of the original and other validation studies may have been caused by the variation in ages between their samples versus the sample used here. Those other analyses included children as young as one year old [26] and children over the age of seven [14,20], unlike the current study where the children age ranges from two to six. In terms of gender, a significant difference between boys and girls in eating behaviour was only found in FR: boys scored higher than girls (mean 2.2 (SD 1.1) versus 1.9 (SD 0.9), *p* = 0.04). This is in line with the outcome of the Chinese version of the CEBQ [27], which suggests that boys seem to be more interested in food than girls.

Cross-sectional associations between children’s BMI z-score and eating behaviours showed positive and strong associations between childhood obesity with ‘food approach’ scales FR and EF, which is consistent with the majority of previous studies [15,22,24,28,36]. Those studies suggest that children with greater BMI are highly responsive to environmental food cues. It was interesting to note that ‘food avoidant’ scales SR and SE had significant negative associations with children’s BMI z-score, similar to the earlier reports [15,22,24,28]. In studies conducted among Singaporean preschool children aged 3, 5 and 6, Quah et al. [38] discovered that the only food avoidance subscale that was substantially linked with lower BMI z-scores at all time points tested was slowness in eating (SE). EUE and FF were observed to have the weakest associations with BMI z-score in previous studies [22,24,28]. Indeed, our results confirm this finding as z-scores showed no association with these ‘food avoidant’ scales. Viana et al. [24] suggested that eating behaviours EUE and FF are not as strongly related to child weight. Similarly, in this study, EOE was found to have positive and significant association with BMI z-score. Other reports whose findings support this notion include the work by Braet and Strien [19], Viana, Sinde and Saxton [24] and Santos et al. [28]. It should be noted that some authors [20,22,23,39] have suggested that EOE onset occurs later in life, and that it might be a relatively atypical eating behaviour for young children. Finally, the ‘food avoidant’ scale DD had no association with adiposity, which reflects previous findings [28].

Interestingly, maternal BMI had a positive and highly significant correlation with child BMI z-scores (r = 0.31, *p* = 0.009) and with ‘food approach’ subscale FR (r = 0.15, *p* = 0.03), whereas there was no effect of paternal BMI on child BMI z-scores and children’s eating behaviours. This outcome reflects a stronger influence of a maternal weight status on obesity in children and it is aligned with several observational studies linking maternal obesity and the development of childhood obesity [40,41]. This may be related to the fact that women oftentimes act as principal caregivers (including by providing meals) and/or that there is an epigenetic link between a child’s propensity to gain weight and maternal adiposity during pregnancy.

Furthermore, children with at least one obese parent or two overweight parents scored slightly higher than children with normal-weight parents or one normal-weight and one overweight parent on FR. This supports the findings of Wardle et al. [42], who reported that preschool children of obese parents are more food-responsive to food cues than children of lean parents. The FR indicates a child’s tendency to eat if given the chance. Consequently, when children are more responsive to food cues, their risk of overeating when food supplies are high increases. Our findings highlight the role of parental influence on food intake and, in a longer term, on body weight. The majority of studies targeting eating behaviours and obesity have been historically carried out in school-aged children and adolescents. However, eating behaviours are to a significant degree shaped even earlier than that, i.e., during the early years, when it is mainly caregivers who provide food to children, create food cues in the immediate environment and model attitudes toward specific diets. For example, parents showing high levels of disinhibited eating, particularly when linked with dietary restraint, have been suggested to foster obesogenic cues [43]. Additionally, young children’s transition of every-day diet is controlled by parents, which points to the formation of children’s eating habits as being started by the end of the preschool period and becoming more stable (i.e., closer to the “adult diet”) from that time on [23]. Indeed, in the cross-sectional study on 1201 children and their parents, Demir and Bektas (2017) found by using the CEBQ and the Parental Feeding Style Questionnaire that children’s eating behaviours and parents’ feeding style impact the risk of obesity [44]. That obesity at an early age is important from the standpoint of long-term metabolic health was already shown in a report from the late 1990s by Whitaker et al. [13], determining that obesity as early as at the age of 3 is a predictor of excessive body weight in adulthood. Hence, altogether, conducting studies on eating behaviours, and defining children’s and parents’ eating habits and attitudes, is crucial for designing interventions that promote healthy eating habits and a healthy weight as well as for understanding the behavioural dynamics of parents-versus-children consumption profiles.

There are a few limitations of the present analysis that need to be mentioned. As in any questionnaire study, body weights and heights were reported by the parents, and medical personnel assisted in determining the height and weight of children only on several occasions. It is, therefore, possible that some of the BMI values may have been under- or overestimated. Secondly, data collection took place at the KFMC, which serves primarily Riyadh and the central province of Saudi Arabia. Follow-up studies incorporating a larger sample size and a comparison between provinces would be of interest, especially in the context of providing a more comprehensive overview representative of the Saudi population rather than only of its subsection. The response rate was comparatively low (59.5%) and more than 65% of parents had university education. This indicates that people with higher levels of education may be more likely to participate in the study. Thirdly, it should also be noted that, since in our cohort, some families reported on more than one child, it would be prudent in the future to consider more in-depth analyses that differentiate between families with a specific number of children of simply limit the number of children one family can report on. Finally, prior to the use of the Saudi CEBQ version, further reliability and validity testing (including the test-retest scenarios as well as construct validity) must be performed in larger samples and in samples that are drawn from non-urban populations, in order to verify the proposed factor structures.

## 5. Conclusions

This study is the first to utilize the CEBQ in the Saudi Arabian context by assessing eating behaviour in Saudi preschool children aged 2–6 years. The analysis revealed an eight-factor solution, confirming the theoretical factor structure. There was no difference in eating behaviours between age groups within the cohort of 2–6-year-old children, but boys were found to score slightly higher than girls on the ‘food approach’ FR subscale. Our results confirm the positive association between children’s obesity and the ‘food approach’ subscales FR, EF and EOE, and the negative association between children’s obesity and the ‘food avoidant’ subscales SR and SE, all of which have been reported previously. Surprisingly, only maternal BMI showed a positive and highly significant correlation with child BMI z-scores. In conclusion, our findings support the use of the CEBQ as a psychometric tool for assessing eating behaviours in Saudi preschool children.

## Figures and Tables

**Table 1:** Demographic and anthropometric characteristics of survey respondents (N = 200).

	N	%
**Children’s Weight Categories**		
Normal	144	72
Overweight	19	9.5
Obese	37	18.5
**Children’s Gender**		
Boys	100	50
Girls	100	50
**Children’s Age Groups**		
2 years	56	28
3 years	40	20
4 years	35	17.5
5–6 years	69	34.5
**Parental Weight Categories**		
Father		
Normal	31	17.7
Overweight	84	48
Obese	60	34.3
Mother		
Normal	48	27.5
Overweight	67	38.2
Obese	60	34.3
**Father and Mother Combined Weight Groups**		
2 overweight or at least 1 obese parent	144	72
2 normal weight or one normal weight and one overweight parent	56	28
**Parental Education**		
Father		
Primary school	7	4
High school	54	30.9
College/University	114	65.1
Mother		
Primary school	8	4.5
High school	50	28.6
College/University	117	66.9
**Completed the Questionnaire**		
Father	19	10.9
Mother	145	82.9
Both parents	11	6.2

**Table 2:** Factor loadings on Direct oblimin rotation of Principal Components Analysis.

Scale Name and Items	Loading	Scale Name and Items	Loading
**Enjoyment of Food EF (Factor 1; 23.05% Variance)**		**Satiety Responsiveness SR (Factor 5; 4.27% Variance)**	
My child loves food	0.63	My child gets full before his/her meal is finished	0.79
My child eats more when s/he is happy	0.51	My child gets full up easily	0.75
My child is interested in food	0.42	My child leaves food on his/her plate at the end of a meal	0.66
My child is always asking for food	0.41	My child cannot eat a meal if s/he has had a snack just before	0.54
My child enjoys a wide variety of foods	0.43	My child decides that s/he doesn’t like a food, even without tasting it	0.43
**Slowness in Eating SE (Factor 2; 10.52% Variance)**		**Food Fussiness FF (Factor 6; 3.89% Variance)**	
My child finishes his/her meal quickly	0.81	My child enjoys tasting new foods	0.76
My child eats slowly	0.7	My child is interested in tasting food s/he hasn’t tasted before	0.72
My child takes more than 30 min to finish a meal	0.68	My child enjoys a wide variety of foods	0.53
My child eats more and more slowly during the course of a meal	0.66	My child is difficult to please with meals	0.49
		My child refuses new foods at first	0.43
**Emotional Under-Eating EUE (Factor 3; 6.79% Variance)**		**Emotional Overeating EOE (Factor 7; 3.43% Variance)**	
My child eats more when s/he is happy	0.43	My child eats more when worried	0.76
My child eats less when upset	0.73	My child eats more when annoyed	0.75
My child eats less when angry	0.69		
My child eats less when s/he is tired	0.53		
My child eats more when anxious	0.52		
**Desire to drink DD (Factor 4; 5.10% variance)**		**Food responsiveness FR (Factor 8; 3.12% variance)**	
If given the chance, my child would always be having a drink	0.91	My child eats less when s/he is tired	0.42
If given the chance, my child would drink continuously throughout the day	0.9	My child is difficult to please with meals	0.48
My child is always asking for a drink	0.78	If given the chance, my child would always have food in his/her mouth	0.65
		Given the choice, my child would eat most of the time	0.52
		My child eats more when s/he has nothing else to do	0.51
		If allowed to, my child would eat too much	0.44

**Table 3:** Factor reliability of the CEBQ (N = 200).

CEBQ Scales	Cronbach’s Alpha	Average Inter-Item Correlation
Emotional overeating EOE	0.69	0.55
Food responsiveness FR	0.76	0.45
Enjoyment of food EF	0.76	0.4
Desire to drink DD	0.8	0.57
Satiety responsiveness SR	0.7	0.33
Slowness in eating SE	0.71	0.45
Emotional under-eating EUE	0.62	0.25
Food fussiness FF	0.68	0.41

**Table 4:** Pearson’s correlations between the CEBQ subscales (N = 200).

	EOE	FR	EF	DD	SR	SE	EUE	FF
Emotional overeating EOE	−							
Food responsiveness FR	0.42 **	−						
Enjoyment of food EF	0.43 **	0.51 **	−					
Desire to drink DD	0.131	0.25 **	0.26 **	−				
Satiety responsiveness SR	−0.21 **	−0.35 **	−0.35 **	−0.033	−			
Slowness in eating SE	−0.11	−0.05	−0.30 **	0.19 **	0.41 **	−		
Emotional under-eating EUE	0.115	0.08	0.27 **	0.14 *	0.17 *	0.16 *	−	
Food fussiness FF	0.25 **	0.37 **	0.55 **	0.21 **	−0.14 *	−0.14 *	0.16 *	−

* Correlation is significant at the 0.05 level (two-tailed). ** Correlation is significant at the 0.01 level (two-tailed). Bold area in the upper-left corner: inter-correlations between ‘food approach’ subscales. Bold area in the bottom right corner: inter-correlations between ‘food avoidant’ subscales.

**Table 5:** Gender and age differences in eating behaviour, eight-factor solution (N = 200).

	Gender			Age				
	Boys (N = 100)	Girls (N = 100)		2 Years (N = 56)	3 Years (N = 40)	4 Years (N = 35)	5–6 Years (N = 69)	
	Mean (SD)	Mean (SD)	*p* ^(a)^	Mean (SD)	Mean (SD)	Mean (SD)	Mean (SD)	*p* ^(b)^
Emotional overeating EOE	1.8 (0.9)	1.7 (0.8)	0.86	1.7 (0.8)	1.7 (0.8)	1.7 (0.9)	1.8 (0.8)	0.69
Food responsiveness FR	2.2 (1.1)	1.9 (0.9)	0.04 *	2.0 (1.0)	2.0 (0.9)	1.9 (0.9)	2.2 (1.1)	0.52
Enjoyment of food EF	3 (0.7)	2.9 (0.9)	0.23	2.9 (0.9)	2.8 (0.7)	2.7 (0.7)	3.1 (0.9)	0.21
Desire to drink DD	3.4 (1.0)	3.2 (1.1)	0.15	3.3 (1.0)	3.5 (1.1)	3.3 (1.1)	3.3 (1.1)	0.85
Satiety responsiveness SR	3.7 (0.8)	3.7 (0.7)	0.86	3.7 (0.7)	3.6 (0.7)	4 (0.9)	3.6 (0.8)	0.11
Slowness in eating SE	3.3 (1.1)	3.3 (1.1)	0.95	3.2 (1.0)	3.3 (1.0)	3.4 (1.1)	3.3 (1.1)	0.82
Emotional under-eating EUE	3.5 (0.9)	3.5 (0.8)	0.81	3.4 (0.9)	3.3 (0.9)	3.4 (0.8)	3.7 (0.9)	0.11
Food fussiness FF	2.7 (1.0)	2.5 (0.9)	0.09	2.8 (1.0)	2.5 (0.9)	2.4 (1.1)	2.7 (0.9)	0.26

^(a)^ *p*-value from *t*-test. ^(b)^ *p*-value from one-way ANOVA. * *p*-value < 0.05.

**Table 6:** The differences in children’s eating behaviour by parental combined weight groups.

	Group 1 ^(a)^ (N = 56)	Group 2 ^(b)^ (N = 144)	*p*-Value ^(c)^
	Mean (SD)	Mean (SD)	
Emotional overeating EOE	1.7 (0.8)	1.8 (0.8)	0.55
Food responsiveness FR	**1.8 (0.8)**	**2.1 (1.0)**	0.02 *
Enjoyment of food EF	2.9 (0.8)	3.0 (0.8)	0.43
Desire to drink DD	3.3 (1.1)	3.3 (1.1)	0.88
Satiety responsiveness SR	3.7 (0.7)	3.7 (0.8)	0.65
Slowness in eating SE	3.3 (1.1)	3.3 (1.1)	0.89
Emotional under-eating EUE	3.5 (0.8)	3.5 (0.9)	0.98
Food fussiness FF	2.5 (0.9)	2.6 (1.0)	0.35

^(^^a)^ Group 1: two normal–weight parents or one normal–weight and one overweight parent; ^(b)^ Group 2: at least one obese parent or two overweight parents; ^(c)^ *p*-value from *t*-test. * *p*-value < 0.05.

**Table 7:** Hierarchical linear regression analyses for BMI z-scores on CEBQ subscales.

Eating Behaviours	Mean	Standard Error (SD)	Standardised β	95% CI for Standardised β		*p*-Value
				Lower Bound	Upper Bound	
‘Food Approach’ Scales						
Emotional overeating EOE	1.75	0.058	0.017	−0.168	0.213	0.818
Food responsiveness FR	2.05	0.070	0.114	−0.062	0.303	0.193
Enjoyment of food EF	2.93	0.059	0.101	−0.124	0.376	0.320
Desire to drink DD	3.34	0.076	0.050	−0.090	0.187	0.492
‘Food Avoidant’ scales						
Satiety responsiveness SR	3.70	0.056	−0.080	−0.314	0.105	0.326
Slowness in eating SE	3.31	0.076	−0.103	−0.253	0.054	0.201
Emotional under-eating EUE	3.49	0.061	0.017	−0.154	0.196	0.814
Food fussiness FF	2.64	0.068	−0.071	−0.248	0.095	0.379

**Table 8:** Correlations between CEBQ scales, BMI Z-score, maternal BMI and paternal BMI.

	BMI Z-Score		Maternal BMI		Paternal BMI	
	R	*p*-Value	R	*p*-Value	R	*p*-Value
BMI Z-score	-	-	0.31 **	**0.009**	0.03	0.62
Emotional overeating EOE	0.16 *	**0.02**	0.08	0.24	0.01	0.99
Food responsiveness FR	0.23 **	**0.001**	0.15 *	**0.03**	0.04	0.52
Enjoyment of food EF	0.24 **	**0.001**	0.08	0.27	0.03	0.64
Desire to drink DD	0.11	0.16	0.09	0.22	−0.08	0.23
Satiety responsiveness SR	−0.17 *	**0.016**	0.08	0.28	−0.05	0.47
Slowness in eating SE	−0.16 *	**0.019**	−0.02	0.74	−0.05	0.45
Emotional under-eating EUE	0.04	0.58	0.02	0.76	−0.03	0.68
Food fussiness FF	0.09	0.21	0.08	0.62	0.03	0.67

* Correlation is significant at the 0.05 level (two-tailed). ** Correlation is significant at the 0.01 level (two-tailed).

## Data Availability

The datasets used and analyzed in the current study are available from the corresponding author on reasonable request.

## Abbreviations

CEBQ: Children’s Eating Behaviour Questionnaire; EF: Enjoyment of food; FR: Food responsiveness; EOE: Emotional overeating; EUE: emotional undereating; DD: Desire to drink; SR: Satiety responsiveness; SE: Slowness in eating; FF: Food fussiness; KFMC: King Fahad Medical City.
